# Surgery

**Published:** 1877-09

**Authors:** 


### II. Surgery.

A Family of Bleeders.—Lossen (*Deutsche Zeitschr. f. Chir.* Band, v. 3).—The family Mampel, of Kirchheim, near Heidelberg, can be traced back to the last century. The originators of this line were married in 1798, both of healthy parentage, in the best of health. They had six sons and four daughters; of the former three were bleeders. In the following generation there were thirty-two children belonging to two of the daughters; of these, thirteen were troubled with the hemorrhagic diathesis. In the third generation there was only one case. All those afflicted were male children, and in appearance did not differ from the rest. *The children of the bleeders remained unaffected. The disease was inherited entirely from the female members, who themselves, however, were not troubled with hemorrhagic diathesis.* During three generations the family counted one hundred individuals, of which seventeen were afflicted with the hemorrhagic diathesis, and nine died of profuse hemorrhage. The hemorrhage occurred from slight external wounds, or from internal, subcutaneous and intermuscular wounds, and sometimes from simple contusions. Hemorrhages also occurred from the gums, the nasal and bronchial mucous membrane, and the mucous membrane of the stomach and bladder, and in the joints hemorrhage frequently took place.

Of the nine fatal cases of hemorrhage, six occurred from simple wounds, one was subcutaneous, one occurred from the lungs, and one from the stomach.

THE MOST FREQUENT CAUSE OF THE RETENTION OF URINE IN OLD PEOPLE.—Prof. W. Busch (*Langenbeck's Archiv Bd.* xx.—3).—The sphincter of the bladder, and the internal orifice of the urethra are very near each other in boyhood, therefore, the the pressure of the urine against the lower part of the bladder finds an easy outlet through the internal orifice. This explains very clearly and easily the enuresis nocturna of children. At puberty the prostate begins to increase in size, and the sphincter is then caused to recede, and a greater force is necessary to expel the urine. If the prostate becomes hypertrophied, the part above the caput gallinaginis becomes enlarged mostly, so that the enlargement encroaches on the bladder, the internal orifice of the urethra is moved toward the bladder, and upward so as to occupy the highest point of the prostatic tumor. It no longer forms the lowest part of the apparatus, but is surrounded on all sides by portions of the bladder, particularly posteriorly, where the bladder has a tendency to assume a ventricular form. When the bladder contracts the whole pressure is exerted against the prostatic enlargements, causing the lobes to approach each other, which in turn are pressed against the interior orifice of the urethra, thus closing it tighter instead of opening it.

This, according to the author's opinion, is more frequently the cause of retention than the enlargement of the middle lobe of the prostate and its action as a valve in the urethra, as is frequently described.

The author's opinion is further strengthened by the fact, that persons suffering from enlarged prostates are not able to expel their water while standing, but they can do so by bending over so as to bring the lowest part of the bladder on a level with the internal orifice.

The autopsy of an old man further confirms the author's opinion. Some time before his death he was unable to pass water, and was obliged to use the catheter. It was found that the prostate gland was not enlarged, but that the bladder immediately behind the prostate had assumed this diverticular form. Now, when the bladder was filled and force was used, this diverticulum was filled up and the prostate was pressed against the urethra as above described, and prevented the escape of urine.

Struma Pulsans Acuta.—Prof. Lucke (*Deutsche Zeitschrift f. Chir.* Band. vi.). The author objects to using the terms, pulsating goitre, and aneurismal goitre, synonymously. Aneurismal goitre as described by Virchow—where the arteries are dilated—has never been observed by the author.

The greatest number of pulsating goitrès are not aneurismal in the full sense of the word. They present the clinical symptoms of aneurism; compressibility, pulsation, and murmur—the same as the pulsating sarcoma; but the dilatation of the arteries is nothing permanent as in an aneurism.

Pulsating goitres occur mostly in youthful individuals. The author thinks they have no connection whatever with Graves' disease.

In 1876 Strassburg was visited by a goitre epidemic, and during this visitation L. observed many cases of pulsating goitres. This pulsating goitre occurs only in the most acute cases, and in the majority of cases preceding strumous diathesis could not be discovered. This acute swelling of the thyroid gland occurs sometimes with such rapidity as to produce the same symptoms as the acute inflammation of the glands; dyspnœa, tracheal stridor and consecutive cyanosis, in consequence of the obstructions to venous circulation. Headache pharyngeal difficulties, etc. These symptoms appear with different degrees of severity. Sometimes these symptoms disappear and the patient recovers, or the pulsations cease, and an ordinary strumous swelling remains.

This complaint is susecptible to treatment; application externally or parenchymatous injections, generally of iodine, being sufficient.

The acute pulsating struma is a tumor not circumscribed and consists, in a majority of cases, of the whole gland. It is soft and disappears under gradual pressure. By palpation the pulsation is very evident. Pulsation is more or less visible, it is most apparent in those cases where there is the greatest dyspnœa; pulsation is also great in the neighboring carotid.

The author is of the opinion that the pulsating struma is but a stage in the development of the acute goitre, and that if the disease persists it will finally form a common goitre, and not an aneurismal tumor. This form of pulsating tumor shows itself mostly in epidemics of acute goitre. It occurs mostly in young people up to the age of 17 years.

The author says there must first be a thorough infection by the goitre miasma, and following this comes the acute dilatation of the capillaries and obstruction to the circulation of the thyroid artery, and in some measure the carotid. He further says that there is considerable analogy between the pulsating struma and pulsating sarcoma as regards the dilatation of the capillaries.

To Prevent the Entrance of Liquids into the Eustachian Tube.—E. Zaufal. (*Monatsschrift für Ohrenheilk.* xi, 4.) In the Präger Med. Wochenschrift Prof. Z. discusses the various measures which have been observed, in order to avoid the unpleasant consequences, that have been known to sometimes arise from the entrance of liquid medicines into the Eustachian tube. He comes to the conclusion that on the one side we can not successfully treat the diseases of the nasal and nasopharyngeal cavities without the use of medicinal fluids, and on the other side all the safeguards so far employed, can not always prevent the fluid from entering the Eustachian tube. Z. now recommends a maneuver which, he claims, will most assuredly render that accident impossible. Standing behind the patient the surgeon, with two fingers, shall push the soft palate boldly upward toward the tubal orifices while liquids are injected into the nostrils. The procedure is painless and not unpleasant, *if only the pressure upon the velum palati be strong.* A slight and slow manipulation occasions a repeated retching, but a strong, bold push causes just one contraction of the pharyngeal muscles that even assists to tightly close the Eustachian tube. Experiments on anatomical preparations as well as the examination with his naso-pharyngeal speculum, have convinced Z. that the tubal orifice is mechanically blocked up by the soft palate whenever the latter is moved upwards, either by the action of its levator as in phonation or by a pressure from below as by the surgeon's fingers.

THE NASO-PHARYNGEAL SPECULUM.—E. Zaufal. (*Monatsschrift fur Ohrenheilk.* xi, 4.) Prof. Z. has recently introduced a funnel-shaped speculum for the direct inspection of the naso-pharyngeal space. These specula come in sets, varying from 9 to 11 centimetres in length, and from 4 to 8 millimetres in width. They are introduced through the inferior or middle nasal meatus, and allow a direct illumination by a reflector, of the posterior nasal and naso-pharyngeal cavities. By means of these specula, Z. was able to diagnose pathological conditions of the naso-pharyngeal cavity in many cases, where without them a correct diagnosis could not be made. But these specula also simplify and facilitate the application of medicine, and the performance of the most frequent operations in the naso-pharyngeal space. The application oftenest called for in that cavity is the cauterization either of ulcers of different kinds, or of hypertrophied tissue around the Eustachian orifice, or of enlarged follicles, or of polypoid vegetations, or of the whole tumefied mucous membrane. Pushing the speculum in so far that its end fairly encircles the spot which is to be cauterized, the surgeon can be perfectly certain of the extent of his application, no matter whether he uses the pure or mitigated caustic, or chromic acid, or the electro-cautery, (in the latter case the speculum must be of hard rubber, which requires a stronger illumination). No other mode of cauterizing is so simple, so painless, so convenient and so efficient as the cauterization by means of the naso-pharyngeal speculum. Another operation in which the naso-pharyngeal speculum can be of great value, is the removal of retro-pharyngeal polypi, by means of the cold or heated wire snare. The difficulty of this operation consists in nicely adjusting the snare around the basis of the tumor so that it will not slip. Now, in the first place, the inspection through the speculum will complete or correct our diagnosis as to the actual site of the basis of the tumor, then when the wire is applied from the mouth, we can examine through the speculum as to its proper application, carefully adjust it, and by means of a proper tenaculum prevent its slipping off. Z. reports three cases to substantiate his remarks, and to illustrate the working of his speculum.

Urethrotomy in a Girl of 9 Years. M. Périer. (*L' Union Médicale*, No. 32, 1877.) M. Périer read a report to the *Sociéte de Chirugie* of a case of urethrotomy, by M. Lemay, upon a little girl of 9 years, who had introduced a needle within the urethra. The needle, escaping from the hands of the child, had penetrated the bladder, and had given origin to a calculus, which interfered with the functions of the organ. The operation allowed extraction of the calculus, which had the needle for a centre, and weighed 15 grammes, (231 grains); a cure followed the operation. The needle had remained in the bladder one month, according to the assertion of the little girl.

The following discussion took place:

M. Théophile Anger offered some remarks upon lithotomy in women for the extraction of vesical calculi. He has twice performed urethrotomy in cases of this kind. In the first case it was a woman of 45 years, who had a stone in the bladder for two years. The stone was small, but occasioned incessant pain. The extraction, by means of urethrotomy, was followed by a rapid cure. M. Anger has since seen the patient, who has had no incontinence of urine.

Last year, M. Anger operated in the same manner upon a young lady of 18 years, who had an immense calculus, which gave her intolerable pain. He succeeded in making, after chloroforming, a large incision, with a probe-pointed bistoury, which gave rise to a considerable loss of blood. The patient got well, but for several months had incontinence of the urine, which had now almost entirely disappeared.

Upon the whole, M. Anger believes that urethrotomy gives better results than vaginal lithotomy.

M. Paulet understands how one may hesitate between urethrotomy and vaginal lithotomy in the case of an adult woman. But it is different in the case of a child, and the choice is not always possible. Some time since, a little girl was brought to him, 12 years of age, but who appeared to be no more than 8 years. She had a stone in the bladder. The question was how to get it away. There was no room by way of the vagina, and vaginal lithotomy was impossible. M. Paulet tried lithotrity, but it was impossible to crush the stone, even with the lithoclast of M. Guyon. He decided to try urethrotomy. A superior incision, made with a probe-pointed bistoury, allowed the introduction of the forceps and extraction of the calculus. There was incontinence for 15 days.

M. Verneuil said that lithotrity in women should be renounced only when its impossibility is evident. After lithotrity, he places the vaginal operation in the first rank, except in children. For very large calculi there remains supra-pubic lithotomy. Cases of incontinence of urine after urethrotomy are not rare. M. Verneuil cites the case of a little girl, upon whom urethrotomy was practiced without success; the stone would not pass; supra-pubic lithotomy allowed the extraction of the stone. The child got well, but incontinence remained, as a result of the urethral incision. Vaginal lithotomy rarely causes a permanent fistula.

To sum up, according to M. Verneuil, urethrotomy should be the exceptional method; he rejects equally forcible dilatation of the urethra. He prefers lithotrity, and if that is inapplicable, the sub-pubic or the vaginal operation.

M. Tillaux wished that this question of lithotomy in women might be cleared up. Malgaigne rejects absolutely the urethral operation, because of the incontinence of urine, and advises the vaginal operation. On the other hand, M. Reliquet declares himself absolutely in favor of urethrotomy, and rejects the vaginal operation.

According to Reliquet, incontinence of urine after urethrotomy depends upon dilatation of the vesical neck without previously chloroforming the patient; the vesical sphincter is then torn, while this accident is not to be feared with chloroform.

M. Verneuil gives the first place to lithotrity in women, when it is possible. According to M. Tillaux, this operation would be less favorable in women than in men. Lithotrity requires the crushing of the stone in a bladder filled with fluid. Now, the liquid does not remain in the female bladder; it comes out with the instrument, and one must work in an empty bladder. M. Tillaux concluded by saying that it was necessary to review the question of lithotomy in women.

M. Launelongue thinks that many surgeons perform lithotrity in the empty bladder, and that it is easier than with fluid in it. The bladder in women is more tolerant than in men, except, however, in young girls. According to important documents collected in the thesis of M. Hybord, lithotrity in women, compared with lithotomy, is an easy operation, not dangerous, and should be practised whenever possible.

Between vaginal lithotomy and urethrotomy, M. Launelongue pronounces himself for the former; it favors healing when disease of this organ complicates calculus.

M. Duplay said that in America, vaginal lithotomy is a current operation, and gives good results. He thinks, with M. Tillaux, that lithotrity is difficult in women, and he regards, above all, lithotrity in the empty bladder as a surgical heresy.

M. Duprès declares that urethrotomy is a bad operation, because it gives no more space than dilatation of the urethra. For himself, he prefers dilatation when haste is required, and lithotrity when one may go more slowly about it. In the latter case, he does not think it more useful to make injections into the bladder, but it is necessary at least, that there should be a little urine present.

M. Marjolin has observed that in little children the bladder is very intolerant, and keeps with difficulty, liquids injected into it. He has noticed, moreover, that calculi are rare among girls; he has seen but one in 18 years.

M. Leon Le Fort said that Thompson advises emptying the bladder first, and then to inject 150 to 180 grammes of liquid. There would be no danger, according to him, in performing lithotrity in the empty bladder.

M. Verneuil this morning performed lithotrity in a woman, or attempted it, rather, but did not remove the stone. He introduced the lithotrite, and moved it easily about the bladder, which was empty, without causing pain. But it is not always so. Certain patients have energetic contractions which do not permit manipulation with the lithotite. It becomes necessary then to inject with precaution, a certain quantity of fluid. It is generally well to have liquid in the bladder.

M. Trelat, when he has a lithortrity to perform commences by emptying the bladder, then injects a moderate quantity of liquid. According to him, it is working blindly to operate without knowing whether or not there is liquid in the bladder.

M. Marc Sée remembers that Nélaton used to empty the bladder, and then inject a certain quantity of water before operating.

M. Périer, the reporter, said that he had only in view, in his report, calculi in girls. In the report of operations made at St. Bartholomew's Hospital, London, by Walch-Hall, it is shown that lithotrity in young girls is injurious. Walch-Hall first dilates the urethra; if the stone is large, he has recourse to the sub-pubic operation; if it is of medium size, he tries vaginal lithotomy. M. Périer believes that incontinence of the urine is less frequent than M. Verneuil supposes after urethrotomy. L. W. C.

A New Method of Curing Popliteal Aneurisms.—Martin Burke, M. D.—(*N. Y. Medical Journal*, June, 1877.—There has recently been introduced into the surgical wards of

Bellevue Hospital a novel method of treating Popliteal Aneurisms, which has thus far proved successful in its application. It consists in the employment of a conical shot-bag suspended from a height by a rope, the apex of which cone presses upon the femoral artery in Scarpa's space and causes all pulsation to cease in the aneurism below. The shot-bag is made of canvas in the form of a flattened cone, its apex measuring one inch in diameter. A rounded piece of cork or india rubber is accurately fitted to the inside of the apex of the cone; a hollow rod of bamboo reaches down to and rests upon the rubber or cork, being held directly in the centre of the cone, while the shot is filled around it to the requisite weight, being about twelve pounds. A cover of canvas is tightly stitched over the base, in the centre of which is an aperture for the passage of the rod. A stout wire hook is fastened to the centre of the base of the cone, to the rod as it emerges from it, and the apparatus is then ready for suspension. To suspend it a pully is driven into the ceiling through which is passed a rope, one end of which is directly attached to the wire hook in the shot-bag, having first been passed through rings attached to the bamboo rod to keep it in place. To the free end of the rope is attached a hollow rubber tubing, to give elasticity to the apparatus, to the extremity of which is attached a hook which connects with the hook in the centre of the base of the shot-bag by means of a linked chain. The amount of pressure is regulated by lifting or lowering the weight in the links of the chain. With the apex of the cone resting on the femoral artery the pressure is carefully regulated until pulsation ceases in the aneurism, no greater pressure being allowed than is necessary to produce this effect.

Three cases are reported as having been successfully treated by this method. The minimum time of application required to produce cessation of the pulsation in the aneurism, was six days, but for security the apparatus was allowed to remain some days longer. The maximim time was sixteen days. The patient suffers but little pain and but little inconvenience. There was no excoriation at point of pressure. A suggestion is put forth that not only for popliteal aneurism, but for aneurisms of other arteries and also for some cases of secondary hemorrhage, with certain modifications, this apparatus may be successfully applied.

